# Effects of post-mortem storage conditions of bovine epididymides on sperm characteristics: investigating a tool for preservation of sperm from endangered species

**DOI:** 10.1093/conphys/cow069

**Published:** 2016-12-29

**Authors:** Julie Strand, Mette M. Ragborg, Hanne S. Pedersen, Torsten N. Kristensen, Cino Pertoldi, Henrik Callesen

**Affiliations:** 1 Section of Biology and Environmental Science, Department of Chemistry and Bioscience, Aalborg University, Fredrik Bajers Vej 7H, DK-9220 Aalborg, Denmark; 2 Randers Regnskov, Tørvebryggen 11, 8900 Randers, Denmark; 3 Department of Animal Science, Aarhus University, Blichers Allé 20, DK-8830 Tjele, Denmark; 4 Aalborg Zoo, Mølleparkvej 63, 9000 Aalborg, Denmark

**Keywords:** Cryopreservation, epididymal spermatozoa, genetic diversity, *in vitro* embryo production.

## Abstract

The aim of this study was to establish and validate a reliable and efficient protocol for the recovery and cryopreservation of epididymal spermatozoa used for *in vitro* fertilization, using bulls of two different age classes. Testicles from 26 (37–51 weeks old, group 1) and 19 (52–115 weeks old, group 2) Danish Holstein bulls were collected after slaughter and stored at 5°C. After 0, 24 or 48 h, epididymides were isolated and spermatozoa collected. Assessments included spermatozoal motility, viability and morphology before and after cryopreservation and *in vitro* embryo production. Results showed that live spermatozoa can be collected from epididymides of bulls after their death. Storage of the testicles at 5°C for 24 h followed by cryopreservation of recovered epididymal spermatozoa resulted in 21% (group 1) and 31% (group 2) blastocysts produced *in vitro.* These results illustrate that epididymal spermatozoa recovered from testicles kept in specific conditions can be used to preserve genetic material from endangered and threatened species or populations in nature as well as in domestic and zoo animals.

## Abstract (precis)

To preserve genetic diversity in threatened populations of domestic and wild animals, cryopreservation combined with reproductive technologies are useful. Here, we present a reliable and efficient protocol enabling preservation of epididymal sperm using domestic cattle as a model species. We propose that this technique has great potential in conservation of threatened species because it can be used to rescue genetic variation in the event of unexpected death, castration or in situations where obtaining an ejaculate is not feasible.

## Introduction

The ability to preserve genetic material is an important tool in conserving genetic variation in endangered populations and species. Biobanks play an integral part in worldwide conservation efforts, in both domesticated and wild species, to counteract the loss of genetic diversity. Furthermore, material in animal biobanks is useful for various types of research, such as cryobiology, reduction of inbreeding, genomic selection studies, assessment of genetic distances and disease genetics (Blackburn, 2012; Groeneveld *et al*., 2016).

When working with domesticated animals, zoo animals and wildlife populations of threatened animals, the number of breeding males is often limited to a few superior animals. Many populations show harem structures with highly skewed mating ratios, and managing a large number of mature breeding males can be problematic from an economic or practical point of view (Sánchez *et al*., 2003; Sørensen *et al*., 2005; Maksudov *et al*., 2008). For breeding and preservation of genetic material, spermatozoa are normally collected through ejaculation, but this is not possible in all species or in every situation, such as in instances of unexpected death or after castration. Furthermore, animals in captivity face various issues, such as space limitations, skewed sex ratios and surplus animals, which leads to euthanization of a number of animals every year. Taken together, several animals die without being used for breeding purposes and thus represent an unexploited source of genetic material (Maksudov *et al*., 2008). Therefore, suitable protocols for recovery of spermatozoa after the animal's death will enable us to exploit and preserve genetic material by cryopreservation of spermatozoa or *in vitro* production of embryos.

Protocols to be used for preservation of genetic material must be developed and practice maintained to be able to react rapidly and competently when needed. Using material from endangered species to develop suitable preservation protocols is a challenge because of the typical low number of animals available for experimentation, so using farm animals such as the bovid is a well-suited model to obtain knowledge that can aid the development of protocols for other species. Recovery of epididymal spermatozoa has been described as a possible method for conserving genetic variation in species such as Sumatran rhinoceros (O'Brien and Roth, 2000), sheep (Kaabi *et al*., 2003), marmosets, baboons and chimpanzees (O'Brien *et al*., 2003), giant pandas (Pérez-Garnelo *et al*., 2004), goats (Santiago-Moreno *et al*., 2006), rhesus monkeys (Dong *et al*., 2008), black and white tegus (Young *et al*., 2013) and saltwater crocodiles (Johnston *et al*., 2014). In these studies, different practical conditions for handling the testicles (storage time and temperature between death and spermatozoa collection) and different approaches to obtain the spermatozoa (slicing, cutting, opening and flushing) have been used. In all studies, evaluation of the protocol included collection efficiency, viability of the spermatozoa after cryopreservation and, in some instances, pregnancy rates after artificial insemination. In none of these studies, however, have the spermatozoa been used for *in vitro* fertilization (IVF), which is relevant for preservation of genetic material and for evaluation of the fertilizing capacity of spermatozoa after *in vitro* handling. In contast, such studies have been carried out in bovids, but they are few and have resulted in fairly low *in vitro* embryo blastocyst rates [6% (James, 2004); 13% (Martins *et al*., 2009); 12% (Chaveiro *et al*., 2015); and 3–16% (Lopes *et al*., 2015)]. Furthermore, no particular information has been provided relating to the importance of the male's age with respect to the spermatozoa. The aim of this study was to establish and validate a protocol for the recovery and cryopreservation of epididymal spermatozoa used for IVF, using bulls of two different age classes.

## Materials and methods

### Collection of testicles

Testicles from 26 younger (37–51 weeks, group 1) and 19 older (52–115 weeks, group 2) Danish Holstein bulls were collected after slaughter (Danish Crown, Aalborg, Denmark) from February to April 2015. The testicles were removed 15–30 min after slaughter and transported at 18–20°C for ~1 h to the laboratory. From each bull, one testicle was processed at this time (control group T0), whereas the other testicle was stored at 5°C in a plastic bag and processed after 24 h (T24; 14 from group 1 and 10 from group 2) or 48 h (T48; 12 from group 1 and nine from group 2).

### Testicle preparation, spermatozoa recovery and quality assessment

All chemicals were purchased from Sigma–Aldrich Corp. (St Louis, MO, USA) except when otherwise indicated. From each testicle, serous coverings and connective tissue were removed (Turri *et al*., 2012) before the epididymis and ductus deferens were isolated. An incision was made between the cauda and corpus epididymis, and spermatozoa were recovered using the retrograde flushing method (Martinez-Pastor *et al*., 2005). Briefly, the lumen of the ductus deferens was cannulated with a 27 gauge needle and flushed in a retrograde direction from the ductus deferens through the cauda epididymis with 3 ml of 32°C warm Sperm-TALP (Table 1). Immediately after recovery, 10 µl of the sperm sample was diluted with Sperm-TALP (dilution factor from ×10 to ×1000) and applied to a haemocytometer (Neubauer, 717820, Brand, Germany) to determine the concentration of spermatozoa and total motile and immotile cells (Atiq *et al*., 2011). The rest of the sperm sample was used for freezing as described later.

**Table 1:** Composition of media used for sperm holding and washing (Sperm-TALP), *in vitro* fertilization (IVF) and *in vitro* embryo culture (IVC), together with the information on the Sigma catalogue number (Cat. no.)

Ingredients	Sperm-TALP	IVF	IVC	Cat. no.
NaCl	5.8 g/l	6.7 g/l	6.3 g/l	S5886
KCl	0.2 g/l	0.2 g/l	0.5 g/l	S5405
NaH_2_PO_4_	0.04 g/l	0.04 g/l		S5011
CaCl_2_.2H_2_O	0.3 g/l	0.3 g/l	0.3 g/l	C7902
MgCl_2_	0.2 g/l	0.1 g/l		M2393
Lactic acid	3.7 ml/l	1.9 ml/l		L4263
Hepes	1.4 g/l			H3784
Hepes-acid	1.1 g/l			H4034
NaHCO_3_	2.1 g/l	2.1 g/l	2.1 g/l	S4019
Pyruvic acid	110 mg/l	0.02 g/l		P3662
Gentamicin	100 mg/l			G1294
Heparin		5 IU/ml		H3149
Sodium disulfite		0.04 µg/ml		S9000
Penicillamine		4.4 µg/ml		P4875
Hypotaurine		0.16 µg/ml		H1384
Epinephrine		0.27 µg/ml		E4250
KH_2_PO_4_			0.2 g/l	P5655
MgSO_4_			0.2 g/l	M2643
Sodium lactate solution			0.6 ml/ml	L4263
Sodium pyruvate			0.08 g/l	P3662
Citric acid			0.1 g/l	C3434
Myo-inositol			0.5 g/l	I7508
Basal Medium Eagle amino acids			30 µl/ml	B6766
Minimum Essential Medium amino acids			10 µl/ml	M7145
l-Glutamine			0.03 g/l	G8540
Gentamicin			0.05 g/l	G1264
Cattle serum			5%	
Dissolved in H_2_O				W1503
Osmolarity	280 ± 8 mosmol/l	280 ± 8 mosmol/l		

Spermatozoa viability and morphology were assessed by analysing eosin–nigrosin (SVS-010, Sperm VitalStain™; Nidacon, Mölndal, Sweden) stained and fixed samples (Bakst and Cecil, 1997). The stain was prepared according to the manufacturer's protocol; 10 µl of the sperm sample was mixed with 10 µl eosin–nigrosin stain for 30 s before each smear was prepared and left to air dry. Each smear was then assessed using light microscopy (Standard lab 06, 380078-0030; Zeiss, West Germany) with a ×400 oil-immersion objective (143108, Flour 40, 1.30 Oil; Nikon). All spermatozoa in a field of view were evaluated, and five fields of view in total were assessed to obtain at least 200 spermatozoa. Spermatozoa that did not take up stain (white) were counted as live membrane-intact cells, whereas spermatozoa with detectable eosin (pink) were counted as dead membrane-damaged cells. The following morphological categories were recorded as described by Blom (1983): tail coilings, deformations, abnormal heads (small, giant) and tail segment missing (Turri *et al*., 2012, 2013). The percentage of each abnormality was calculated.

### Freezing and thawing of epididymal spermatozoa

Freshly prepared OPTIXcell extender (024385; IMV Technologies, l'Aigle, France) was diluted 1:2 and incubated at 32°C for 10 min before being used within 6 h. From the sperm sample, 400 µl was diluted 1:1 with OPTIXcell extender and mixed gently, before incubation at 32°C for 10 min. The sperm sample was then further diluted with OPTIXcell extender to achieve a final concentration of 80 × 10^6^ spermatozoa/ml, before being incubated at 25°C for 10 min. Subsequently, the sample was stored at 5°C for 4 h before being loaded into 0.25 ml straws (340704; Kruuse, Marslev, Denmark). The straws were transferred to a metal rack located 4 cm above liquid nitrogen for 15 min, then plunged and finally stored in liquid nitrogen for 3–4 weeks.

### Quality assessment after freezing

Straws were thawed in water at 35–37°C for 2 min, and the spermatozoa concentration, total motility, viability and morphology were assessed as described above.

### *In vitro* production of embryos (i.e. maturation, fertilization and culture)

Ovaries were collected at the Danish Crown slaughterhouse and transported to the laboratory within 2–4 h in 0.9% NaCl solution (Pharmacia AS, Copenhagen, Denmark) at 32–36°C. Cumulus–oocyte complexes were aspirated from 2–6 mm follicles with a 19 gauge needle. The cumulus–oocyte complexes were collected and washed once in Hepes-buffered Medium 199 (M0650) supplemented with 30 IU/ml heparin (from stock 5000 IU/ml, LEO Chemical Factory, Ballerup, Denmark), 10 µl/ml amphotericin (A2942) and 2% cattle serum (CS; Danish Veterinary Institute, DTU, Frederiksberg, Denmark), 10 µl/ml amphotericin (A2942) and 2% cattle serum (CS; Danish Veterinary Institute, DTU, Frederiksberg, Denmark). Cumulus–oocyte complexes with a minimum of three to four cumulus cell layers were selected for *in vitro* maturation and transferred in groups of 25 per well of four-well dishes (176740; Thermo Fisher Scientific, Roskilde, Denmark) containing 400 µl *in vitro* maturation medium [bicarbonate-buffered Medium 199 (M2154) supplemented with 10 IU/ml equine chorionic gonadotrophin and 5 IU/ml human chorionic gonadotrophin (constituents of Suigonan Q; Intervet Scandinavia, Skovlunde, Denmark), 117 mg/l l-glutamine (G8540), 50 µg/ml gentamicin (G1264) and 15% cattle serum] and overlaid with 400 µl oil (M5310). Immature cumulus–oocyte complexes were incubated for 23–25 h at 38.5°C in humidified air supplemented with 5% CO_2_.

Owing to limited resources, only bulls at T24 were used for IVF (we randomly selected eight bulls from group 1 and eight bulls from group 2; we used 75 oocytes per bull). Straws from each bull were thawed in warm water (35–37°C) for 2 min, and the spermatozoa were washed with 2 ml Sperm-TALP by centrifugation for 10 min at 277***g***. The supernatant was removed and the pellet resuspended in 2 ml Sperm-TALP. This procedure was repeated twice. After the third spin, the stock volume was reduced to 100 µl in all samples.

While the spermatozoa were being washed, the oocytes were transferred in 50 µl *in vitro* maturation medium to the IVF wells of four-well dishes containing 400 µl IVF medium (Table 1) and overlaid with 400 µl oil. Mature cumulus–oocyte complexes were transferred to IVF wells without washing, so the IVF medium contained ~1% cattle serum originating from the *in vitro* maturation medium. Spermatozoa from each bull were added to the IVF media at a final concentration of 2 × 10^6^ spermatozoa/ml; this time was designated as Day 0. Oocytes and spermatozoa were incubated for 20–22 h at 38.5°C with air enriched with 5% CO_2_.

Presumptive zygotes were vortexed at 250*g* for 45 s in 0.2 ml Hepes-buffered Medium 199 with 5% cattle serum in a 6 ml tube (734-0436, VWR, Radnor, PA, USA). The zygotes were recovered and washed in *in vitro* culture medium (Table 1) before being transferred in groups of 25 per well to a four-well dish containing 400 µl *in vitro* culture medium overlaid with 400 µl oil. The embryos were cultured at 38.5°C in an atmosphere of 5% CO_2_, 5% O_2_ and 90% N with 95% relative humidity (Galaxy R CO_2_ incubator; RS Biotech). Embryos were evaluated on Days 2 and 7 for cleavage and blastocyst rates, respectively. As a control for each experimental round, IVF was performed using cryopreserved ejaculated spermatozoa from an 18-month-old bull with known *in vitro* fertility.

### Statistical analysis

Statistical analyses were carried out using JMP 11 (JMP statistical discovery™; SAS Institute Inc.), using both parametric and non-parametric analyses because of the small sample size, where a normal distribution can be difficult to achieve. The Shapiro–Wilk test for normal distribution indicated that some of the analysed variables were normally distributed, whereas others were not. Transformation did not increase normality or homogeneity of variances for those traits that did not fulfil these criteria, and for this reason untransformed data were analysed.

A two-way analysis of variance (ANOVA) was used to analyse the effect of storage time (S = T0, T24 or T48), the age of the bull (A = group 1 or group 2) and their interaction (S × A) on pre-freeze and post-thaw bull epididymal spermatozoa samples. The significance of any differences was analysed by the Wilcoxon test, which compares the median of the distributions (Zar, 2010).

## Results

### General patterns

Epididymal spermatozoa were successfully collected from all 90 testicles with the retrograde flushing method. The total number of spermatozoa was 9306 × 10^6^ [95% CI: 469.1 × 10^6^ to 1392.9 × 10^6^ (group 1)] and 13 960 × 10^6^ [95% CI: 872.2 × 10^6^ to 191.9 × 10^6^ (group 2); *P* > 0.05].

### Effect of age and storage time on quality

In general, motility and viability tended to increase from 0 to 24 h storage, followed by a decrease after 48 h storage (Table 2A). Considering the two age groups, motility was higher in group 2 compared with group 1 bulls.

**Table 2:** Effect of storage time (S), age (A) and the interaction between age and storage on motility, viability and abnormal spermatozoa rate of epididymal bull spermatozoa before freezing (**A**) and after freezing (**B**) (two-way ANOVA; n.s. = *P* > 0.05, *P < 0.05, **P < 0.01 and ***P < 0.001)

(A)															
	Storage time (S)						Storage time (S)			Age (A)		Two-way ANOVA			
	T0		T24		T48										
Age (A)	Group 1	Group 2	Group 1	Group 2	Group 1	Group 2	T0	T24	T48	Group 1	Group 2	RMSE	S	A	S × A
Testicles (*n*)	26	19	14	10	12	9	45	24	21	52	38				
Motility (%)	47.9	60.9	60.7	63.9	42.8	55.9	54.4	62.3	49.4	50.5	60.2	14.6	*	**	n.s.
Viability (%)	48.5	59.6	61.5	59.4	50.2	61.5	54.0	60.5	55.9	53.4	60.2	15.7	n.s.	n.s.	n.s.
Abnormal sperm (%)	8.8	7.4	9.2	9.1	7.3	7.6	8.1	9.2	7.5	8.4	8.1	2.5	n.s.	n.s.	n.s.

(B)												
	Storage time (S)				Storage time (S)		Age (A)		Two-way ANOVA			
	T24 a.f.		T48 a.f.									
Age (A)	Group 1	Group 2	Group 1	Group 2	T24 a.f.	T48 a.f.	Group 1	Group 2	RMSE	S	A	S × A
Testicles (*n*)	14	10	12	9	24	21	26	19				
Motility (%)	43.6	51.9	24.9	36.8	47.7	30.8	34.2	44.3	10.8	***	**	n.s.
Viability (%)	44.9	48.1	37.0	37.8	46.5	37.4	40.9	43.0	12.5	*	n.s.	n.s.
Abnormal sperm (%)	7.3	8.4	8.9	8.5	7.9	8.7	8.1	8.5	2.7	n.s.	n.s.	n.s.

Abbreviations: RMSE, root mean square error; T0, testicles processed after 0 h; T24, testicles processed after 24 h; T48, testicles processed after 48 h.

When combining the two treatment groups, age and storage time (Table 2A), the general pattern was clearly illustrated by the young bulls (group 1). Motility increased after 24 h and decreased after 48 h of storage; for viability and abnormal spermatozoa, only a tendency for a similar change was observed. Spermatozoa from the older bulls (group 2) did not show similar changes for motility and viability (Table 2A).

### Effect of age and storage time on freezability

In general, freezing and thawing resulted in a decrease in motility and viability (Table 3), especially for the spermatozoa stored for 48 h. When comparing bulls of different ages (group 1 or 2), the effect of freezing and thawing was found to be highest on the motility of spermatozoa from the younger bulls after storage for both 24 and 48 h (Table 2B).

**Table 3:** Wilcoxon test on dependent data for changes in motility and viability after freezing of epididymal bull spermatozoa stored for either 24 or 48 h

Motility or viability	Before freezing	After freezing	Wilcoxon test
	Mean (95% CI) (*n*)	Mean (95% CI) (*n*)	Significance
Motility T24 vs. motility T24 a.f.	62.1 (51.8–72.4) (24)	47.0 (37.5–56.5) (24)	***
Viability T24 vs. viability T24 a.f.	60.6 (46.7–74.5) (24)	46.3 (37.0–55.6) (24)	**
Motility T48 vs. motility T48 a.f.	48.4 (34.4–62.4) (21)	26.9 (16.9–36.9) (21)	***
Viability T48 vs. viability T48 a.f.	55.0 (39.8–70.2) (21)	37.3 (26.0–48.6) (21)	***

Values are means with 95% confidence intervals (CI) and sample size (*n*). Abbreviation: a.f., after freezing. **P < 0.01 and ***P < 0.001.

### Effect of age on *in vitro* fertilization

The frozen–thawed spermatozoa from testicles stored for 24 h produced cleavage and blastocyst rates that tended to be lower for group 1 [40.5 (95% CI: −5.89 to 86.93, *n* = 8) and 21.0 (95% CI: −0.01 to 41.97, *n* = 8), respectively] compared with group 2 [57.9 (95% CI: 19.95–95.81, *n* = 8) and 30.5 (95% CI: 10.90–50.11, *n* = 8), respectively]. Group 1 had significantly lower (*P* < 0.05) cleavage and blastocyst rates than the reference bull [78.2 (95% CI: 61.18–95.21, *n* = 13) and 35.9 (95% CI: 25.60–50.14, *n* = 13), respectively], whereas only the cleavage rate in group 2 was significantly different from the reference bull (*P* < 0.05).

## Discussion

The protocol used in this study was efficient in generating viable and motile epididymal bull spermatozoa, which could be cryopreserved and subsequently used successfully for IVF with higher developmental rates than described previously.

The method for retrograde flushing resulted in a satisfying recovery of spermatozoa, as found previously in different species (Sankai *et al*., 2001; Kaabi *et al*., 2003; Martinez-Pastor *et al*., 2005; Lone *et al*., 2011; Turri *et al*., 2013). Motility and viability at 0 h tended to be lower compared with results after 24 and 48 h storage. This could indicate variation between the testicles from one individual, but could also be caused by the fact that T0 testicles were not refrigerated at 5°C but kept for up to 3 h at room temperature before being processed. Refrigeration of the epididymis has resulted in sustained motility and viability (Lone *et al*., 2011; Turri *et al*., 2013; Takeo *et al*., 2014), probably as a result of the reduced metabolic rate of spermatozoa at 5°C (Salamon and Maxwell, 2000). When stored for 48 h, the motility and viability declined, illustrating a storage effect also found by Martins *et al*. (2009) in bovids, but total motility was the parameter most affected by storage time also in other species (Sankai *et al*., 2001; Kaabi *et al*., 2003; Martinez-Pastor *et al*., 2005).

This study is the first to assess the effect of the age of the bull on epididymal spermatozoa performance. A general finding was that bulls that had just reached sexual maturity (group 1) had lower spermatozoal quality at recovery. This is in agreement with studies of changes in semen characteristics around puberty (39–41 weeks of age, for Holstein bulls; Amann, 1983), showing an increase in the percentage of morphologically normal and live spermatozoa as well as in cell motility and numbers as the bulls mature (Evans *et al*., 1995; Dance *et al*., 2016). Also, spermatozoa from younger bulls in this study demonstrated less robustness to the different challenges (storage and cryopreservation), resulting in lower rates of both IVF and embryo development. Cryopreservation is known to have damaging effects on spermatozoa, e.g. reduced motility, DNA fragmentation and lower fertilization rate (Critser *et al*., 1987; Wakayama and Yanagimachi, 1998; Takeda *et al*., 2015). Specific proteins (e.g. protamine) are important for minimizing spermatozoal DNA strand breaks, and in bovids a positive correlation between age and protamine content has been proposed (Fortes *et al*., 2014), making this spermatozoal parameter interesting for future studies comparing spermatozoal quality and fertility in younger and older bulls.

Our results showed that refrigeration followed by cryopreservation of epididymal spermatozoa from both group 1 and group 2 bulls allowed production of embryos *in vitro* with satisfying rates, especially from group 2 bulls. Other studies have demonstrated lower developmental rates (James, 2004; Martins *et al*., 2009; Chaveiro *et al*., 2015; Lopes *et al*., 2015), and this may be caused by differences in methods for transport and storage, e.g. a 24 h transportation time of the oocytes (James, 2004), different extenders for spermatozoal handling (Lopes *et al*., 2015) or other freezing and thawing rates (Chaveiro *et al*., 2015).

In conclusion, cooling of testicles shortly after recovery is important to obtain the best-quality spermatozoa. For preservation of the spermatozoa, storage should preferably be performed for no longer than 24 h, although there is a reasonable flexibility up to 48 h after the testicles are obtained. The younger bulls showed less robust semen, giving less flexibility in the practical work, and emphasizing the importance of working faster with younger males. When done, the embryo production rate after IVF is acceptable, and the method can be used for producing embryos for transfer. We expect the protocol to be fairly simple to adjust to other species, producing a practical tool to save genetic material from various endangered populations and species. The genetic material can thereby be reintroduced into various populations that suffer from genetic consequences of small population size, such as inbreeding depression and loss of genetic variation owing to genetic drift (Maksudov *et al*., 2008; Browne *et al*., 2011; Purdy *et al*., 2016).
